# Black Phosphorus: Critical Review and Potential for Water Splitting Photocatalyst

**DOI:** 10.3390/nano6110194

**Published:** 2016-10-29

**Authors:** Tae Hyung Lee, Soo Young Kim, Ho Won Jang

**Affiliations:** 1 Department of Materials Science and Engineering, Research Institute of Advanced Materials, Seoul National University, Seoul 08826, Korea; sunshinety@snu.ac.kr; 2 School of Chemical Engineering and Materials Science, Chung-Ang University, Seoul 06974, Korea; sooyoungkim@cau.ac.kr

**Keywords:** black phosphorus, 2 dimensional (2D) material, optoelectronic, photocatalyst, solar water splitting

## Abstract

A century after its first synthesis in 1914, black phosphorus has been attracting significant attention as a promising two-dimensional material in recent years due to its unique properties. Nowadays, with the development of its exfoliation method, there are extensive applications of black phosphorus in transistors, batteries and optoelectronics. Though, because of its hardship in mass production and stability problems, the potential of the black phosphorus in various fields is left unexplored. Here, we provide a comprehensive review of crystal structure, electronic, optical properties and synthesis of black phosphorus. Recent research works about the applications of black phosphorus is summarized. Among them, the possibility of black phosphorous as a solar water splitting photocatalyst is mainly discussed and the feasible novel structure of photocatalysts based on black phosphorous is proposed.

## 1. Introduction

Recently, there has been growing attention to two-dimensional materials. Graphene, two-dimensional chalcogenides and two-dimensional oxides are known as two-dimensional materials family [1]. These two-dimensional materials contribute to various research areas, especially in transistors, optoelectronics, energy storage system and so on. Though, existing two-dimensional materials have properties that restrict their applications. In the case of graphene, its absence of the band gap energy reduces its applicability in semiconducting and optoelectronic devices. The two-dimensional layer form of silicon, which is called as silicene, has the main issue on its environmental stability. Transition metal dichalcogenides (TMDCs) have direct band gap and strong light absorption that can be utilized in the optoelectronic devices. However, the Mo- and W-based TMDCs that are mainly used have relatively high band gap energies. Thus, they are only proper for the absorption of limited portion of visible lights for optoelectronic applications. Furthermore, TMDCs have relatively low mobilities when compared to graphene. These drawbacks hinder the improvement of device performance using TMDCs for electronic applications and broad applications, which require different band gap range. Therefore, there is growing needs for new two-dimensional materials that can overcome drawbacks mentioned above.

Phosphorus is well known for a commonly used material in explosives and matches. Its structural instability and toxicity have been keeping physicists and chemists from applying outstanding electronic, optical properties of the black phosphorus in various fields [2,3]. However, recently, almost a century after its first discovery, black phosphorus is rediscovered as a promising two-dimensional material. Due to its distinct properties compared with other two-dimensional materials, black phosphorus is extensively studied in field effect transistors and other electronic devices [4,5,6,7]. In recent years, according to several researches, it is reported that black phosphorus can also be a promising candidate for solar water splitting [8,9,10].

## 2. Crystal Structure and Synthesis

**Figure 1:**
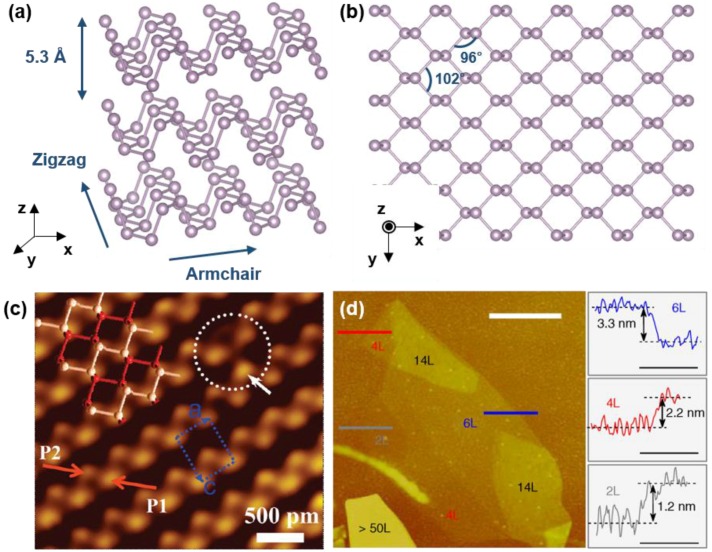
(**a**,**b**) Schematic diagram of the crystal structure of black phosphorus. System is relaxed using density functional theory calculation. (**c**) Close-up showing Scanning Tunneling Microscope (STM) image of the upper atoms of topmost puckered layer of black phosphorus (Reproduced with permission of [14]. Copyright American Chemical Society, 2009). (**d**) Atomic Force Microscopy (AFM) images of black phosphorus flakes. Estimated layer numbers are indicated in the image and line scans are performed along the colored lines in the AFM image (Reproduced with permission of [23]. Copyright Macmillan Publishers Limited, 2014).

Black phosphorus forms a layered structure with single element, phosphorus, just like graphene. However, it has several differences when compared with other layered materials consisting of group IV elements. In its layered structure, phosphorus atoms have five valence electrons which have configuration of 3s^2^3p^3^ valence shell. To build the bond with other atoms, each phosphorus atom goes through hybridization and forms sp^3^ hybridized orbitals. These orbitals make covalent bonding with adjacent four phosphorus atoms and this constructs a puckered structure. In Figure 1c, the upper (white line) and lower (red line) layers of the black phosphorus single layer are visualized and the puckered structure is clearly identified. Because of its puckered structure, a single layer of black phosphorus consists of two atomic layers and includes two kinds of interatomic bonds. The shorter bond with length 0.2224 nm connects phosphorus in the same layer and the longer bond with length 0.2244 nm connects phosphorus at top and bottom in single layer [11,12]. With the schematic diagram of the black phosphorus in Figure 1a, it is possible to confirm its layered structure with space of 5.3 Å. Each layer interacts with other layers with weak van der Waals forces. In addition, there are two distinctive directions at the edge of black phosphorus, zigzag and armchair. These two different directions cause anisotropy in the black phosphorus and this topic will be discussed in latter part of the paper. In Figure 1b, with the top view of the black phosphorus, the hexagonal structure with bond angles 102.09° and 96.34° is shown [11,13].

The crystal structure of black phosphorus can be varied with the pressure. The stable crystalline structure of the phosphorus at 1 bar is orthorhombic and has Cmca (C 2/m 2/c 2_1_/m) space group. This orthorhombic unit cell can be seen in Figure 1c, the portion surrounded by blue dotted lines [14]. If the pressure goes up to 5 GPa, the structure of black phosphorus transforms from orthorhombic to rhombohedral. Again, if the pressure reaches the 10–11 GPa range, black phosphorus finally changes its structure to simple cubic [15,16]. This structural change due to pressure can give an influence to several properties of black phosphorus. Band gap or exciton binding energy varies with pressure and optical property such as luminescence can be also influenced [17,18].

Black phosphorus can be synthesized by a variety of methods. Its first synthesis was done by Bridgeman [19] in 1914, by converting white phosphorus to black phosphorus at 373 K under 1.2 GPa within 5–30 min. In 1981, Maruyama et al. [20] found the method to synthesize black phosphorus in comparatively moderate pressure, by using the solution of white phosphorus and liquid bismuth at 573 K under 0.5 MPa. Different from the synthesis methods using white phosphorus as mentioned, researchers began to select red phosphorus as a raw material for black phosphorus fabrication recently. In 2007, Lange et al. [21] discovered that black phosphorus can be prepared from red phosphorus via the addition of gold, tin and tin (IV) iodide in small amounts under low pressure condition at 873 K. Then, in 2014, Kopf et al. [22] showed that black phosphorus can be grown by a short way transport reaction with red phosphorus, tin and tin (IV) iodide additives. The mixture of Sn, SnI_4_ and red phosphorus was located in the furnace at the temperature of 650 °C. Then the mixture was cooled down to the 550 °C during 7.5 h for the synthesis of black phosphorus. In contrast to the previous method by Lange et al., they succeeded to reduce the amount of side phases such as Au_3_SnP_7_, AuSn or Au_2_P_3_. Exfoliation to fabricate phosphorene, few layer phosphorus, is also being implemented in various methods. In Figure 1d, it is possible to identify that mechanical exfoliation can produce black phosphorus range from 50 layers to even two layers. In several researches, by mechanical exfoliation, phosphorene is fabricated for electronic devices such as Field Effect Transistors (FETs) [23]. Although mechanical exfoliation is quite useful for fundamental research, the large-scale production of phosphorene is still difficult. For large-scale production, several researches performed liquid phase exfoliation. Brent et al. in 2014 and Guo et al. [24] in 2015 introduced a basic *N*-methyl-2-pyrrolidone (NMP) liquid exfoliation method, producing phosphorene by 4 h sonication of bulk black phosphorus in NaOH NMP solution. In addition, Yasaei et al. [25] suggested dimethylformamide and dimethyl sulfoxide as an appropriate solvent for a liquid exfoliation method. 

## 3. Electronic Structure

**Figure 2:**
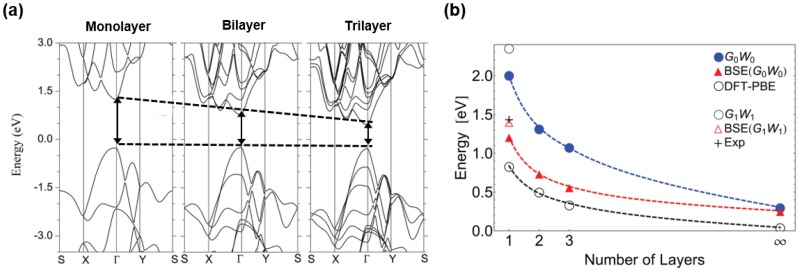
Electronic structure of the black phosphorus is calculated using first principles calculation. (**a**) Calculated electronic band structure of monolayer, bilayer and trilayer black phosphorus. Direct band gap of each layer is presented with black arrow at gamma position. The energy is scaled with respect to the Fermi energy (Reproduced with permission of [34]. Copyright IOP Publishing LTD, 2014). (**b**) Band gap with increasing number of layers are calculated by various methods and fitting curves are presented with dashed lines. The experimental value is brought from [38] (Reproduced with permission of [35]. Copyright American Physical Society, 2014).

The electronic structure of black phosphorus has been studied extensively by both in experimental and computational methods [11,26,27,28,29,30,31,32,33]. Figure 2a shows the band structures of the monolayer, bilayer and trilayer of black phosphorus constructed by density functional theory calculation with HSE06 hybrid functional. Compared with other two-dimensional materials, the electronic structure of black phosphorus has some distinct properties. Black phosphorus has a direct band gap at the Γ point of the Brillouin zone regardless of its number of layers. In the case of transition metal dichalcogenides (TMDCs), they have direct band gaps at the K point, only for the single layers and commonly they have indirect band gaps. From band gaps marked with dashed black line in Figure 2a, the dependency of black phosphorus‘ band gap on number of layers is shown [34]. This thickness dependence is clearly identified with calculation data in Figure 2b. To figure out the band gaps of monolayer, bilayer, trilayer and bulk black phosphorus, several calculation methods were employed. From ab initio calculations with the GW approximation, the band gap of black phosphorus varies from 2 eV for monolayer to approximately 0.3 eV for bulk [35]. Thickness dependence of the band gap is due to charge carriers‘ quantum confinement effect, in the out-of-plane direction. TMDCs also have band gaps varying with thickness but this dependence is much stronger in black phosphorus [36]. Beyond its number of layers, there are several more factors that can alter the band gap. Liu et al. [37] demonstrated that by alloying black phosphorus with arsenic, the alloys can have band gaps between the range of 0.15 eV–0.3 eV. Besides alloying, Rodin et al. [18] suggested that strain can induce modification of band gap. By the Density Functional Theory and the Tight-Binding Theory, they predicted the effect of strain on monolayer black phosphorus. As the strain increases, the original direct band gap of black phosphorus changes into an indirect band gap and, at last, it shows the zero band gap of a metal. Liu et al. [38] calculated the strain dependence of the band gap of black phosphorus with HSE06 as well. In case of negative strain, both armchair and zigzag direction strains lead to band gap decrease. On the other hand, when the positive strain is applied, the zigzag direction strain consistently increases the band gap while the band gap abruptly decreases with more than 5% armchair direction strain. Stacking pattern of black phosphorus is another factor that is possible to deal with the band gap. Dai et al. [39] suggested three different stacking models of bilayer black phosphorus. Models are divided into the cases that the top layer is stacked directly on the bottom layer (AA-stacking), the bottom layer is shifted half of the cell in direction of *x* or *y* presented in Figure 1 (AB-stacking), and the top and bottom layers have mirror images of each other (AC-stacking). Resulting from different stackings, calculated band gaps of bilayer black phosphorus have the range of 0.8 eV–1.05 eV. The factors mentioned till now provide the tunability of unconstrained black phosphorus compared with other two-dimensional materials and it allows the broad application of black phosphorus for various purposes. 

Electronic properties of black phosphorus are governed intensely by its carrier mobilities and are closely related to effective mass of the carriers. Qiao et al. [40] predicted the effective mass and mobility of charge carriers with first-principle calculations. Effective masses of both electron and hole decrease when the number of black phosphorus layer increases. Compared with each other, hole has the lower effective mass and, thus, the conductivity of the hole surpasses that of electron, making the hole as the major carrier in black phosphorus. n-type black phosphorus can be achieved by doping. Substituting Te atoms to P atoms let doped black phosphorus have n-type conductivity [41].

Black phosphorus shows superconductivity when high pressure is applied on it. This property is mainly due to the transformation of the structure as mentioned before. After the superconductivity of black phosphorus first discovered by Wittig et al. [42] in 1968, there were several approaches to find out the path to obtain superconductivity. Kawamura et al. [43] achieved superconductivity with two different paths. First, pressure of 15 GPa is applied at room temperature, which makes black phosphorus transform into a simple cubic phase. Then, in a constant pressure, the sample was cooled down to the liquid helium temperature. The transition temperature (6 K) was measured. Another path began with rapid cooling of black phosphorus to 4.5 K without applying pressure. Then, pressure was increased to 30 GPa. The dependence of the transition temperature on pressure showed that transition temperature increases from 4 K to 10.7 K when pressure rises from 11 to 30 GPa. The third path starts with raising pressure to 8.7 GPa until black phosphorus becomes a rhombohedral phase. Then, it was cooled down to 4.5 K and superconductivity was observed at 5.7 K under 9 GPa [44,45]. The appearance of the superconductivity is deeply relevant with the band gap variation with the applied pressure. As mentioned above, black phosphorus transforms into different phases and at last becomes metallinc phase with zero band gap. This shows that all the properties of the material can be abruptly changed under an extremely high pressure. 

## 4. Anisotropy

Anisotropic properties of black phosphorus are mainly due to its crystal structure. The anisotropy of black phosphorus is much stronger than other two-dimensional materials such as graphene or Mo- and W-based TMDCs. There are properties which show anisotropy and they can be divided into mechanical property, optical property and electronic property. Jiang and Park [46] performed first-principles calculations and revealed that black phosphorus has different Young’s modulus value for the armchair direction and the zigzag direction. In single layer black phosphorus, Young’s modulus for the armchair direction has the value of 21.9 N·m^−1^, while the zigzag direction has more than twice larger value, 56.3 N·m^−1^. In the same manner, thermal conductivity shows similar tendency with the study of Lee et al. [47]. They compared the thermal conductivity along zigzag and armchair directions with suspended-pad micro-devices under the condition of steady-state longitudinal heat flow. The anisotropy is clearly distinguished when temperature is above 100 K. 

**Figure 3:**
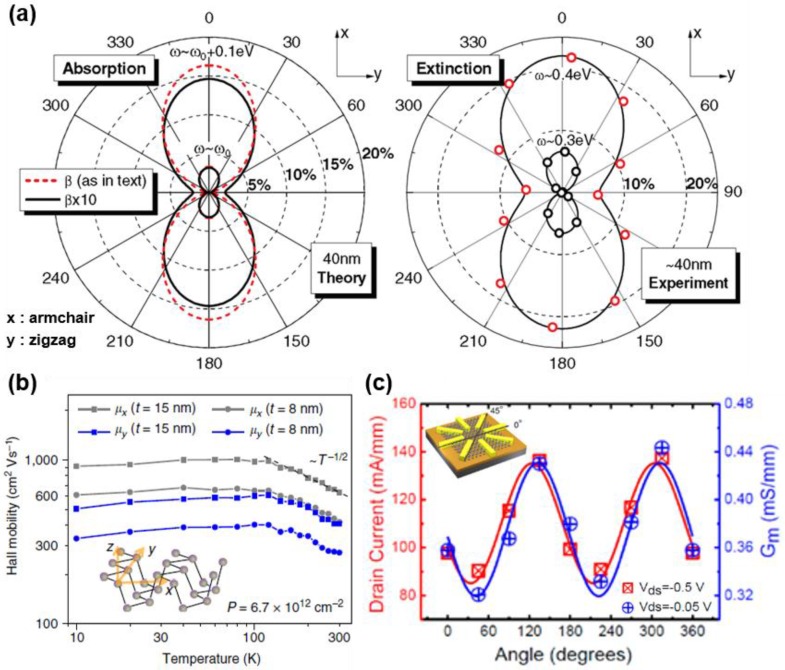
(**a**) Polar representation of the absorption coefficient and experimental extinction spectra of the 40-nm intrinsic black phosphorus film. Absorption coefficient is represented with two different interband coupling strength β which gives different band gap value ω. Extinction spectra are obtained from FTIR spectroscopy with black phosphorus on SiO_2_ substrate (Reproduced with permission of [49]. Copyright American Physical Society, 2014). (**b**) Hall mobility measured along *x* (armchair) and *y* (zigzag) direction on 8 nm and 15 nm black phosphorus film with constant hole doping concentration of 6.7 × 10^12^ cm^−2^. (**c**) Schematic of the device structure for determining the angle-dependent transport behavior and angular dependence of the drain current and the transconductance of a device. Device is composed of electrodes and black phosphorus film with 10 nm thickness (Reproduced with permission of [38]. Copyright American Chemical Society, 2014).

In addition, by solving the phonon Boltzmann transport equation based on first-principles calculations, Qin et al. [48] calculated the thermal conductivity of black phosphorus. At 300 K, the thermal conductivities of zigzag direction and armchair directions are 30.15 Wm^−1^·K^−1^ and 13.65 Wm^−1^·K^−1^, respectively. Figure 3a plots the angular dependency of absorption coefficient and extinction spectra. The plot of absorption coefficient and extinction spectra both shows the dumbbell shape. Along the armchair direction (*x*-axis), black phosphorus represents high absorption and extinction. For zigzag direction, there is not any absorption or extinction relative to its perpendicular direction [49]. These optical property anisotropies are strongly induced by some extrinsic factors, such as structural deformation, corrugations or defects. Figure 3b shows the Hall mobility of the black phosphorus along the armchair (*x*-axis) and zigzag (*y*-axis) directions. For both thicknesses, 8 nm and 15 nm, mobilities along the armchair direction are approximately double compared with those along the zigzag direction. The anisotropic behavior of electric property was also measured in a multi-electrode transistor based on black phosphorus. The schematic in Figure 3c represents that a transistor consisting of Ti/Au contact and few-layer phosphorene was fabricated. Contacts are allocated in round shape with equivalent angular interval, 45 degrees. The maximum drain current at a 30 V back gate bias and a 0.5 V drain bias is displayed in Figure 3c. The maximum drain current shows a sinusoidal-like curve with obvious angle dependent property. The maximum drain current has its minimum value of 85 mA/mm at 45° and 225° and maximum value of 137 mA/mm at 135° and 315° [38].

## 5. Device Applications

Due to the superior electronic properties with some novel properties and recent rediscovery as a promising two-dimensional material, black phosphorus gathers extensive attention with its device application. At the moment, research for its application in areas such as electronics, optoelectronics and batteries is in progress [4,5,6,8,50,51,52,53,54].

**Figure 4:**
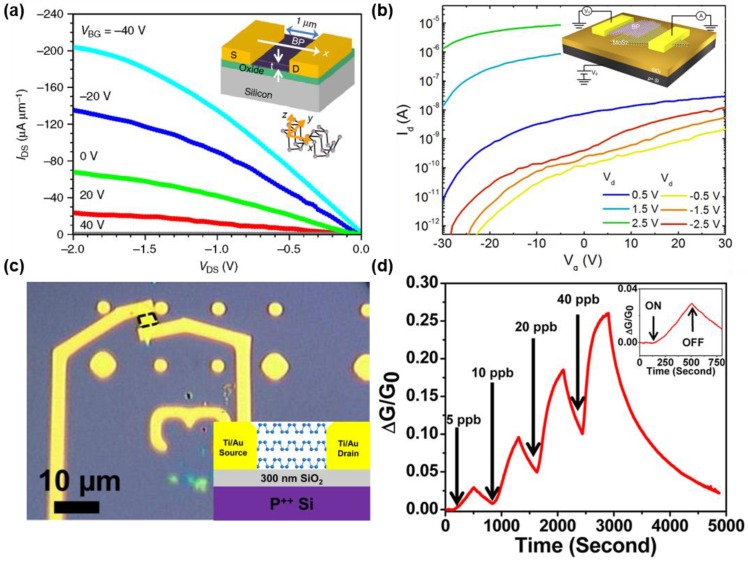
Nanodevices based on black phosphorus. (**a**) Current saturation curve of the black phosphorus field effect transistor along the *x* direction. The channel length of the transistor is 1 μm with 5 nm thickness black phosphorus film (Reproduced with permission of [23]. Copyright Macmillan Publishers Limited, 2014). (**b**) Transfer curve of the p-n diode consist of black phosphorus and MoS_2_ monolayer (Reproduced with permission of [6]. Copyright American Chemical Society, 2014). (**c**) Schematic and images of the multilayer black phosphorus field effect transistor used for chemical sensing. (**d**) NO_2_ gas sensing performance of the black phosphorus field effect transistor. Relative conductance change is represented during 5000 s with different ${\mathrm{NO}}_{2}$ concentration (Reproduced with permission of [7]. Copyright American Chemical Society, 2015).

Transistor is one of the most basic and important applications in electronics. For the outstanding performance of transistor, the channel material should possess high carrier mobility, high on/off ratio and high conductivity with low conductance when it is turned. Other two-dimensional materials such as graphene or TMDCs are suggested as a candidate for the channel material. Graphene has a remarkable carrier mobility from 3000 cm^2^/Vs to 27,000 cm^2^/Vs [55]. However, because of its metallic behavior with its zero band gap, it is impossible to achieve low off-state current. In the case of TMDCs, they possess high on/off ratios but relatively low carrier mobilities, which interfere with enhancing transistor performance. On the other hand, black phosphorus is able to satisfy required properties. Its mobility can reach up to ~1000 cm^2^/Vs when the direction is regulated and high on/off ratios, superior to that of TMDCs, can be achieved [6,29,51,52,53]. The actual performance of FET with black phosphorus is exhibited in Figure 4a. The channel of the transistor is designed along the armchair direction of black phosphorus, which has an excellent electrical property. The source-drain current versus source-drain voltage with back gate bias range from −40 V to 40 V is shown in Figure 4a and, compared with the graphene transistor, it shows much enhanced current saturation behavior [23]. Figure 4b shows a transfer curve of the p-n diode consisting of black phosphorus-monolayer MoS_2_ heterojunction. It is possible to identify the modulation effect of back gate voltage with the transfer curve. With increasing the back gate voltage, forward and reverse currents also increase considerably [6].

The potential of black phosphorus as a gas sensor has been reported in several researches [7,54,56,57]. According to first-principles study based on Density Functional Theory, nitrogen based gases, especially NO or NO_2_ make the strongest binding with black phosphorus. With adsorption of nitrogen based gases, current along the black phosphorus increases while the adsorption of the gas such as NH_3_ reduces current. Most black phosphorus based gas sensors are fabricated as FETs. In Figure 4c, FET used in black phosphorus gas sensor is depicted. With Ti/Au contacts on both sides, black phosphorus is used as the channel material and deposited on the Si/SiO_2_ substrate. Figure 4d represents the sensing behavior of the black phosphorus gas sensor. NO_2_ gas is alternately flowed and blocked, which is expressed as on and off, respectively. The concentration of the flowing NO_2_ gas increases gradually from 5 ppb to 40 ppb. The black phosphorus sensor clearly gives the response to NO_2_ concentration, showing 0.29 for 5 ppb and 0.25 for 40 ppb [7]. Recently, new synthesis method of the few layer black phosphorus for the hydrogen peroxide sensing was demonstrated by Yan et al. [57] With carbon dioxide-assisted synthesis method, the black phosphorus showed a competitive detection limit of 1 × 10^−7^ M for hydrogen peroxide sensing.

## 6. Black Phosphorus as Water Splitting Photocatalyst

As mentioned above, the application of the black phosphorus is mainly concentrated on electronic devices such as FET. On the other hand, its possibility as a water splitting photocatalyst has not been studied much. Recently, several researches adopted black phosphorus as a photocatalyst or photoelectrode [8,9,10]. Zhou et al. [8] proposed TiO_2_, black phosphorus heterostructure for the solar cell and Sa et al. [9] suggested strain engineering of the black phosphorus for its future application as a photocatalyst. 

Commonly, photocatalyst is a substance that accelerates the photoreaction. By absorbing light, it generates electron-hole pair (EHP) and the ability to create EHP determines the photocatalytic activity. Photocatalysis process can be divided into several steps. First, with incident light, solar photons generate the EHP and photocatalyst becomes in excited state. Then generated EHP migrates to the interfaces of solid and water without recombination. At the interface, transferred electrons and holes reduce and oxidize the adsorbed substances if it is favorable in thermodynamic viewpoint. 

In the photocatalytic water splitting, semiconducting materials absorb photons from irradiated sunlight. The semiconducting photoelectrode or photocatalyst absorb photons higher than their band gap energy. The free energy required to convert one mole of H_2_O to H_2_ and 1/2O_2_ under standard condition is ∆G = 273.2 kJ·mol^−1^ and the water splitting process can be demonstrated as Equation (1).

2H_2_O = O_2_ + 2H_2_; ΔG = +273 kJ·mol^−1^ (1)

**Figure 5:**
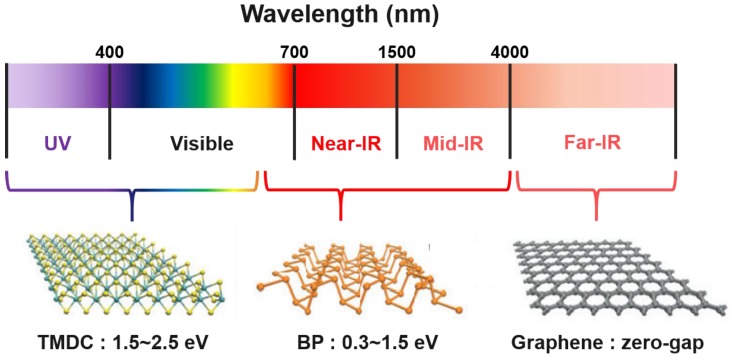
Electromagnetic wave spectrum and band gap range of three types of two-dimensional materials (Reproduced with permission of [61]. Copyright Macmillan Publishers Limited, 2014).

The energy required for the photocatalytic water splitting reaction, ∆G = 273.2 kJ·mol^−1^, corresponds to ∆E° = 1.23 V per electron transferred according to Nernst equation. Thus, for the full water splitting reaction, the conduction band minimum (CBM) should be more negative that reduction potential of water and valence band maximum (VBM) should be more positive than oxidation potential of water. Considering the overpotential arises at solid/liquid interface and kinetic overpotentials, the semiconductor should have higher band gap value than 1.23 V. Heretofore, TMDCs are widely studied as promising materials which can give high efficiencies as photocatalysts, with its moderate band gap and optical properties [58,59,60]. With the band gap range from 1.5 to 2.5 eV, TMDCs mainly absorbs the ultraviolet and the large portion of visible light. Considering that visible light takes the large portion in sunlight, photocatalyst with TMDCs can give high efficiency. Likewise, black phosphorus, as a same two-dimensional material, has similar optical properties and is expected to show efficiency corresponds to that of TMDCs, or even better. In Figure 5, the absorption spectra of three two-dimensional materials, TMDCs, black phosphorus and graphene, are presented [61]. Black phosphorus covers the Near, Mid IR regions and a part of visible lights as a single material. Considering the covering region of the various TMDCs, the tunability of the black phosphorus is remarkable. Even more, the absorption range of black phosphorus can be broadened with several components such as doping, applied strain and different stacking methods [18,37,38,39]. These features therefore raise black phosphorus as a promising candidate for photocatalyst.

**Figure 6:**
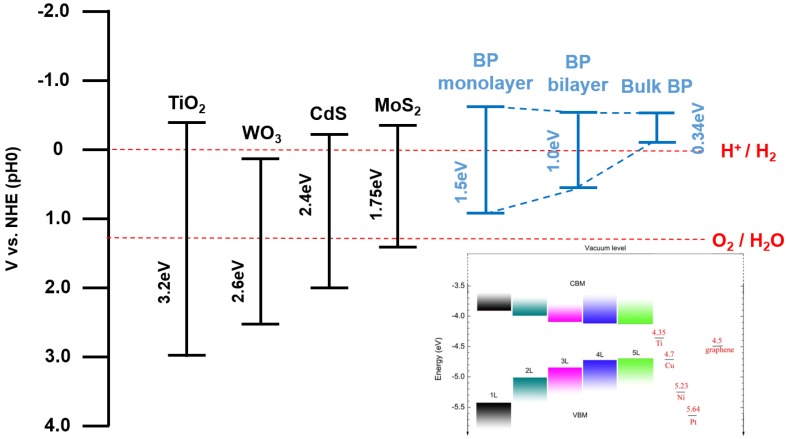
Band edge position of few semiconductors and monolayer, bilayer, bulk black phosphorus using the normal hydrogen electrode (NHE) as a reference. The band edge position of the few layer black phosphorus at vacuum level is also represented from HSE06 calculation (Reproduced with permission of [34]. Copyright Nature Publishing Group, 2014).

For the selection of a material as photocatalyst, it is important to consider the band position of the material as well as band gap. The band gap of black phosphorus varies with its number of layer as mentioned before. In Figure 6, the band gap and band position of monolayer, bilayer, and bulk black phosphorus are presented. The band position of the black phosphorus is calculated with first principles calculation method and it can be identified at the lower right part of the Figure 6. Based on the calculation data, normal hydrogen electrode potential of the black phosphorus is presented in Figure 6, with blue colored range. In the case of bulk black phosphorus, its band position shows that it is improper for water splitting but phosphorene gives a potential. Compared with redox potential of O_2_/H_2_O (1.23 V), the VBM of the monolayer black phosphorus is not more positive, residing at the lower position. On the other hand, the CBM of the monolayer black phosphorus lies below the redox potential of H^+^/H_2_ (0 V), giving more negative potential. These mean that black phosphorus can be used for half water splitting, especially hydrogen evolution reaction (HER). 

## 7. The Stability of the Black Phosphorus

Despite its promising potential as a photocatalyst, the innate instability of black phosphorus hinders its application. Its instability also causes some troubles in other electronic devices such as FETs and it is mainly due to degradation by oxygen, light, water and temperature. The reaction of the phosphorus with oxygen at surface is the most major factor of degradation. The interaction with the oxygen has been established by both calculation and experiment. During interaction, O_2_ molecule easily dissociates at the surface and forms the oxidized surface [62,63,64]. O_2_ molecule approaches to the surface of the phosphorene with perpendicular configuration and after dissociation, oxygen chemisorbed black phosphorus forms. The dissociation energy of the oxygen is exothermic (−4.07 eV) and has a barrier of 0.54 eV [62]. As a result of the reaction, interstitial oxygen can also occur and both interstitial and chemisorbed oxygen atoms deteriorate the properties of black phosphorus devices by initiating degradation. 

The interstitial oxygen can have two metastable bridge-type surface defects, the diagonal bridge configuration and the horizontal bridge configuration, in the phosphorene. In the diagonal bridge configuration, oxygen bonds with two phosphorus atoms at different layers which consist of monolayer. Oxygen in the horizontal configuration, on the other hand, lies on the equivalent height phosphorus atoms and forms bonding with them. These two bridge-type surface defects are developed with positive binding energies. With the oxygen bridge, huge structural deformation occurs and deep donor/acceptor levels are introduced in the gap of black phosphorus. The levels caused by oxygen bridge accelerate the recombination at the surface of black phosphorus [64].

Chemisorbed oxygen which has the dangling configuration on the black phosphorus surface is the most stable configuration compared with other configurations mentioned above, the diagonal and horizontal bridge configurations. Unlike the bridge configurations that oxygen has two bondings with two phosphorus atoms, oxygen in the dangling configuration has bonding with only one phosphorus atom, which is similar with a dangling bond. Because of the difference in electronegativity between oxygen and phosphorus, oxygen adsorbed phosphorus have increased hydrophilicity and this leads to degradation by water molecule. 

The influence of water to degradation of black phosphorus is also as significant as that of oxygen. Commonly, it is well known that black phosphorus has strong affinity for water molecule. There are several researches about humidity impact on the performance of black phosphorus used devices. According to Island et al. [3], when left in the ambient condition, there was 200% increase of volume over several days due to water adsorption. Moreover, the strong dipole-dipole interaction between water molecule and phosphorus causes significant distortion on black phosphorus structure. 

As well as gas molecules like oxygen or water, light and temperature also contribute to the degradation of phosphorene. In the research of Favron et al. [65], with the oxygen and water existent condition, the photoinduced degradation of the phosphorene arises. The oxidation rate is proportional to the oxygen concentration and light intensity. Thermal stability of the two-dimensional black phosphorus is studied by Liu et al. [66] by in-situ scanning/transmission electron microscopy. Annealed with temperatures ranging from 200 °C to 500 °C, the decomposition of black phosphorus is observed at ~400 °C in vacuum. The decomposition initiates with eye shaped crack and at last amorphous red phosphorus remains. 

**Figure 7:**
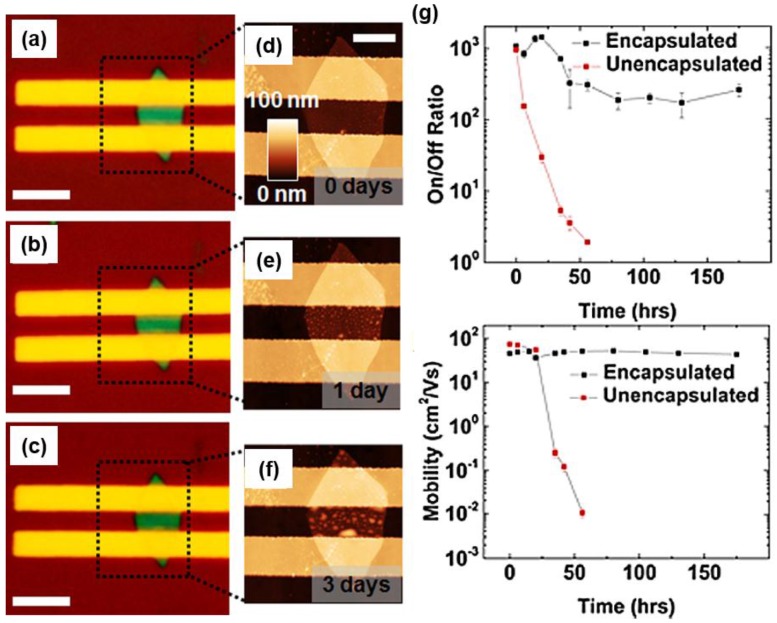
(**a**–**c**) Optical images of the black phosphorus field-effect transistor, uncapsulated. They are left in ambient conditions for 0, one, and three days, in order. The thickness of the flack is approximately 8.9 nm and scale bars are 5 μm. (**d**–**f**) Each corresponds to the AFM height image of (**a**–**c**). The length of the scale bar is 2 μm and height scale is represented in (**d**); (**g**) On/off ratio and hole mobility of the black phosphorus field effect transistor for encapsulated one and unencapsulated one (Reproduced with permission of [67]. Copyright American Chemical Society, 2014).

The factors of degradation mentioned above are fatal obstacle for the application of black phosphorus to devices. Especially, for water splitting photocatalyst, preventing degradations caused by oxygen and water molecules are critical for its performance. In Figure 7a–f, it is possible to identify the progress of the degradation in the ambient condition. As time passes, bubble shaped degradation is created on the surface of black phosphorus layer. The performance deterioration can be seen in the Figure 7g. To prevent detrimental effect of the degradation, Wood et al. [67] made the passivation on the black phosphorus layer. As we can see in Figure 7g, passivated (encapsulated) black phosphorus maintains its properties well compared with uncapsulated one. Thus, it seems obvious that passivation on the black phosphorus layer blocks the degradation by oxygen and water molecule. 

## 8. Black Phosphorus-Based Heterostructure and Its Potential as Photocatalyst

For water splitting, it is important to acquire high solar-to-hydrogen conversion efficiency for development of a low cost strategy. In solar water splitting process, when irradiated photons are absorbed by photocatalyst, EHP are generated. Among the created EHP, some portion of electrons recombine with holes and become extinct. After the recombination, left EHP can take part in redox reactions at the photocatalyst. Thus, to increase solar-to-hydrogen conversion efficiency, suppression of the recombination is essential [68].

The construction of heterostructure can be an effective solution for improving solar-to-hydrogen efficiency of the photocatalyst. First of all, with heterostructure, electrons and holes generated by absorbing photons are separated. Therefore, the recombination of the EHP is suppressed. Furthermore, owing to heterostructure using multiple semiconductors which have different band gaps, it becomes possible for photocatalyst to absorb photons with broader range of wavelength. 

As mentioned above, heterostructure can maximize the potential of black phosphorus photocatalyst, by decreasing the recombination rate. Recently, there are several researches about heterostructure of black phosphorus and other materials. Guo et al. [69] presented the phosphorene and graphene heterostructure as anode materials for rechargeable lithium batteries. Based on first-principles calculations, high mobility and stability of heterostructure anode were identified. Heterosturctures of phosphorene and TMDCs, especially sulfide hybrids, for application in solar cell were also studied. Guo et al. calculated the electronic structure of MoS_2_, WS_2_/Phosphorene with first-principles calculations with the HSE06 computation. The solar cell system consists of multilayer phosphorene and a monolayer MoS_2_ gives a power conversion efficiency (PCE) of 11.5% to 17.5%, the maximum PCE at monolayer phosphorene. Dai et al. [39] constructed the heterostructure of bilayer black phosphorus and MoS_2_, varying the stacking of black phosphorus layer. The combined trilayer builds the type-II heterojunction alignment and PCE is predicted to be around 18%. In addition to managing recombination rate, heterostructures of the black phosphorus with particular materials such as HfO_2_ can slow down the degradation of black phosphorus [70].

Lately the heterostructures of black phosphorus and oxides, especially TiO_2_, were presented as promising materials for solar cell and photocatalyst. Commonly, oxides are not preferred as photocatalyst because of their large band gaps. Due to their large band gaps, oxides mainly absorbs ultraviolet region of the solar spectrum, losing the large portion of the solar energy. Thus, constructing heterostructure with black phosphorus, which absorbs the visible light region, can be an effective solution for drawback of oxide photocatalysts. Zhou et al. [8] constructed the heterostructure by depositing monolayer black phosphorus on the TiO_2_ substrate (110) surface. Constructed heterostructure shows extraordinary charge separation efficiency, which result in high PCE and enhanced photoactivity. Black phosphorus and TiO_2_ hybrid photocatalyst was also presented by Lee et al. [71]. By constructing heterostructure, the photocatalyst can absorb the light range from 250 nm to 1200 nm, covering whole solar spectrum: ultraviolet, visible light, and infrared region. The stability of the black phosphorus and TiO_2_ heterostructure photocatalyst is estimated in the water solution. Unlike the common supposition of black phosphorus’ high affinity to water, constructed heterostructure does not show the oxidized phosphorus, either degradation bubbles. However, it is still uncertain about the stability under photo-illuminated condition and there is possibility of light induced degradation. 

**Figure 8:**
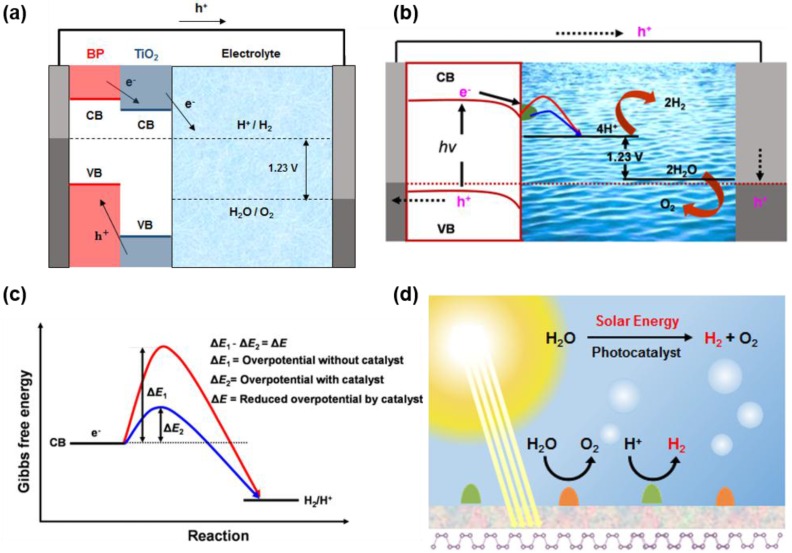
(**a**) Schematic of energy band diagram of the black phosphorus monolayer photocathode with titanium dioxide passivation layer. (**b**) Schematic of the photocathode with p-type semiconductor photoelectrode and catalyst. (**c**) Electron transfer paths for a bare photoelectrode and a photoelectrode with catalyst (Reproduced with permission of [72]. Copyright Springer international Publishing, 2016). (**d**) Photoelectrochemical water splitting using monolayer black phosphorus photocathode with passivation layer and titanium dioxide photocatalyst.

The heterostructure of thin layer black phosphorus and metal oxide, especially TiO_2_, build the band alignment as depicted in Figure 8a. The monolayer black phosphorus has higher CBM and VBM compared with TiO_2_ and this provides the path for electrons to occur hydrogen evolution reaction. With the heterostructure photocatalyst, the overpotential for the electron transfer to solution is reduced extensively. This process is presented in Figure 8b,c as a schematic [72]. Loading catalyst gives the alternative pathway for electron to go over the lower activation energy. In Figure 8d, all the factors mentioned before are put together. The passivation layer is deposited on the monolayer of black phosphorus and TiO_2_ is located above it, just like the heterostructure of Lee et al. [71]. Considering the results of various researches, this form of heterostructure is expected to be a promising candidate for HER photocatalyst. 

## 9. Conclusions

In summary, we have seen fundamental features and interesting properties of black phosphorus. Its extensive tunability of electronic and optical properties, and extraordinary anisotropy are receiving attention among other two-dimensional layered materials. Until now, most researches are concentrated on its application to electronic devices by controlling its band gap and electron mobility. However, there are still many unexplored applications and properties left. Studies about the potential of black phosphorus as a photocatalyst is emerging lately. It is proven that by regulating optical and structural properties of the black phosphorus, it can successfully replace other metal including photocatalysts. Though, as usual, there are some challenges left for the smooth application of black phosphorus to water splitting photocatalyst. Still, there are not enough researches about its hydrophilicity and degradation behavior under illuminated condition. Furthermore, comparatively high EHP recombination rate and alteration of the band edge position caused by tuning band gap may act as an obstacle for its practical use as a photocatalyst. Thus, it is necessary to further develop effective strategies such as a passivation or heterostructure of black phosphorus. On the whole, based on innate superb properties of black phosphorus, many more breakthroughs in its application for water splitting photocatalyst is anticipated in the foreseeable future.
